# The effect of Chinese herbal medicine combined with western medicine on vascular endothelial function for patients with hypertension: Protocol for a systematic review and meta-analysis

**DOI:** 10.1097/MD.0000000000018134

**Published:** 2019-12-10

**Authors:** Weiquan Ren, Jiangquan Liao, Jialiang Chen, Zhonghao Li, Li Huang

**Affiliations:** a Beijing University of Chinese Medicine; b China-Japan Friendship Hospital, Beijing, China.

**Keywords:** hypertension, protocol, systematic review, traditional Chinese medicine

## Abstract

**Background:**

Essential hypertension is one of the most common chronic diseases in the world and a major risk factor for cardiovascular and cerebrovascular diseases. Hypertension often leads to a variety of complications, of which vascular endothelial dysfunction is an important part. Traditional Chinese medicine (TCM) combined with western medicine can significantly improve vascular endothelial function in patients with hypertension, but it has not been systematically evaluated for efficacy and safety of essential hypertension. Therefore, we aim to conduct a systematic review and meta-analysis to evaluate the efficacy and safety of TCM combined with western medicine in improving vascular endothelial function in patients with essential hypertension.

**Methods:**

We will search PubMed, Cochrane Central Register of Controlled Trials (CENTRAL), Excerpta Medica Database (EMBASE), China National Knowledge Infrastructure Database (CNKI), Wanfang Database, China Science Journal Database (VIP Database) and China Biomedical Literature Database (CBM). Clinical trial registrations, potential grey literature, related conference abstracts, and reference lists of identified studies will also be retrieved. The electronic database will be searched for literatures published from the beginning to October 2018. Based on the heterogeneity test, data integration is performed using a fixed effect model or a random effects model. Changes in blood pressure and endothelial function will be assessed as primary outcomes. Drug use, disease progression and adverse events will be assessed as secondary outcomes. RevMan V.5.3.5 will be used for meta-analysis.

**Results:**

This systematic review and meta-analysis will provide high-quality evidence from a variety of aspects, including efficacy, blood pressure, vascular endothelial function and adverse reactions, to assess the efficacy and safety of TCM combined with western medicine in patients with hypertension.

**Conclusion:**

This systematic review will determine whether TCM combined with western medicine provides evidence for effective intervention of vascular endothelial function in patients with essential hypertension.

**Ethics and dissemination:**

This systematic review and meta-analysis of randomized controlled trials does not require ethical recognition, and the results of this paper will be published in an open access, internationally influential academic journal.

**PROSPERO registration number:**

CRD42019140743

## 1 Introduction

Essential hypertension is the most common chronic disease and a major risk factor for cardiovascular disease. The main complications include stroke, myocardial infarction, heart failure, and chronic kidney disease, which not only lead to high disability and mortality, but also require a large amount of health care and social resources. Hypertension has caused a heavy burden on families and national health care, and has become an important public health problem in China.^[1–3]^ Studies have shown that vascular endothelium plays a fundamental role in regulating vascular tension and structure, and endothelial dysfunction and resulting structural changes may be the cause of adverse effects of hypertension.^[4]^ The primary goal of hypertension treatment is to reduce the overall risk of heart, brain, kidney and vascular complications development, and death. Common antihypertensive drugs include calcium channel blockers, angiotensin converting enzyme inhibitors, angiotensin receptor blockers, diuretics, and β-blockers.^[5]^ However, due to cost, adverse reactions, complications, etc, nearly half of patients are unable to effectively control blood pressure through drugs.^[6]^

Traditional Chinese medicine (TCM) is an important part of complementary and alternative medicine. Although the mechanism is not clear, it has been widely accepted in China and applied in clinical practice.^[7]^ A large number of studies have shown that TCM decoction, Tai Chi, and acupuncture can effectively treat hypertension.^[8–11]^ Nowadays, the treatment of hypertension with TCM combined with western medicine is more and more concerned by clinical doctors and patients, and improvement of the vascular endothelial function of hypertensive patients can improve its prognosis. There is no clinical evidence for the improvement of vascular endothelial function in patients with hypertension in comparison with TCM and western medicine. Therefore, we have developed a protocol for systematic evaluation and meta-analysis to evaluate the efficacy and safety of TCM combined with western medicine in improving vascular endothelium in patients with hypertension, and can provide reference applications for clinical use.

## 2 Methods

### 2.1 Study registration

The protocol of this review has been registered with the international Prospective Register of Systematic Reviews (PROSPERO; registration number: CRD42019140743) and has been reported in accordance with the Preferred Reporting Items for Systematic Reviews and Meta-analyses guidelines.^[12]^

### 2.2 Inclusion criteria for study selection

#### 2.2.1 Types of studies

Inclusion criteria: Randomized controlled trials (RCTs) that use Chinese herbal medicine (CHM) combined with western medicine to treat hypertensive patients, regardless of blinding, will be included in this study.

Exclusion criteria: non-RCTs; participants do not meet the inclusion criteria; studies which have been published repeatedly; therapeutic measures failing to meet the predetermined inclusion criteria; control group not western medicine; (6) no blood pressure data for extraction. Language and time will not be restricted to minimize publication bias.

#### 2.2.2 Types of participants

Inclusion criteria: patients aged 18 and over, but under 80 years of age; patients with a diagnosis of hypertension that is consistent with past guideline definitions (SBP ≥ 140 mm Hg or DBP ≥ 90 mm Hg).

Exclusion criteria: Subjects with hypertension and other serious cardiovascular diseases, hepatic failure, and renal failure; subjects with secondary hypertension; subjects with gestational hypertension; and subjects with isolated systolic hypertension.

#### 2.2.3 Types of interventions

This study focuses on the RCTs of hypertension with the therapy of CHM combined with western medicine, and the results will provide advice and consultation for clinicians. All types of CHM, combined with western medicine, with no limitations on the dose, the method of dosing, the composition of the formula, or the duration of administration. Studies that with combination therapy fail to objectively evaluate the efficacy and safety of CHM will be excluded. Studies of control groups were treated with western medicine.

#### 2.2.4 Types of outcome measures

All types of CHM, combined with western medicine, with no limitations on the dose, the method of dosing, the composition of the formula, or the duration of administration.

##### 2.2.4.1 Primary outcomes

The primary outcomes will include SBP, DBP, 24 hour SBP, and 24 hour DBP. The therapeutic effectiveness is determined according to the 2010 Chinese guidelines for the management of hypertension^[13]^: markedly effective = diastolic blood pressure decreased >20 mm Hg (1 mm Hg = 0.133 kPa) or decreased <10 mm Hg, but has reached the normal range of blood pressure; effective = diastolic blood pressure decreased >10 to 19 mm Hg or decreased <10 mm Hg, but has not reached the normal range the normal range of blood pressure; invalid = did not meet the above criteria. Meanwhile, nitric oxide, endothelin-1, flow-mediated dilation, vascular endothelial growth factor, high-sensitive C-reactive protein, angiotensin II, von Willebrand factor, and transforming growth factor β-1 are also included.

##### 2.2.4.2 Secondary outcomes

The secondary outcomes are any adverse events (nausea, vomiting, diarrhea, and so on).

### 2.3 Search methods for identification of studies

#### 2.3.1 Electronic searches

Seven electronic databases including: PubMed, Embase, CENTRAL, CBM, CNKI, Wanfang Data, and VIP database. These databases were searched from inception to April 2019 for the relevant RCTs of CHM for essential hypertension. Search terms to be used will include essential hypertension, CHM, and RCTs. The search strategy for Medline will be searched via PubMed and is shown in Table 1; other electronic databases will also be searched based on this strategy.

**Table 1:**
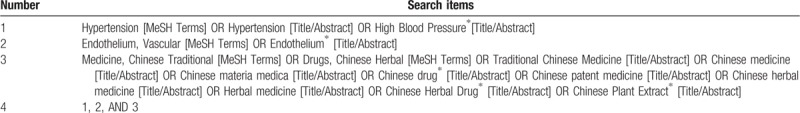
Search strategy for Medline.

#### 2.3.2 Searching other resources

A reference list of research and systematic reviews will be reviewed and retrieved for additional testing. Potential gray literature will be searched in OpenGrey.eu. We will search for relevant conference abstracts to find eligible trials. In addition, we will search the World Health Organization International Clinical Trial Registration Platform (ICTRP) and the clinical trial website ClinicalTrials.gov for all new comments related to this topic.

#### 2.3.3 Data collection and analysis

##### 2.3.3.1 Selection of studies

**Figure 1:**
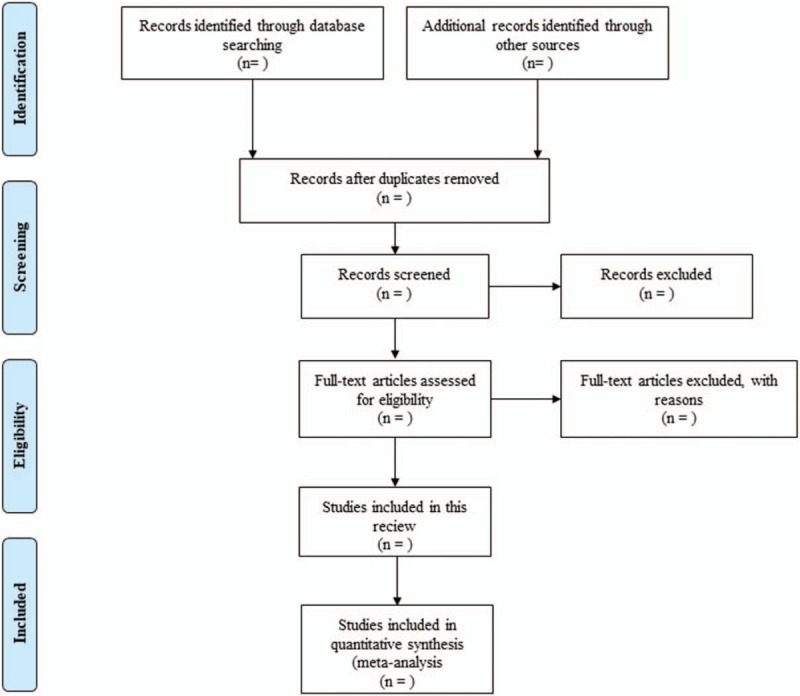
Flow diagram of the literature searching and study selection.

According to the Cochrane Collaboration System Evaluator's Manual (5.1.0), the basic process of the included literatures will be determined. An excel spreadsheet will be used to record the study name, author, year, database, and whether the study met eligibility criteria and should be included in the review. During abstract screening and full-text evaluation, reasons for inclusion and exclusion are also recorded in a spreadsheet. The records in this spreadsheet will be used to generate the Preferred Reporting Items for Systematic Reviews and Meta-Analyses flowchart (Fig. 1). Two reviews will carry out all the procedures independently and complete cross check. If any disagreement occurs, the 3rd author will be invited to assist in the discussion and make a decision.

##### 2.3.3.2 Data extraction and management

Before data extraction, we will make a standard data collection form. Two reviewers will independently extract data from selected studies and complete a data collection form. Differences and uncertainties will be resolved by consensus between the 2 review authors or by a 3rd author to make a final decision. We will extract the following data:

1. General information: 1st author, title, journal, type of publication, year of publication, country, source of funding.
2. Methods: study design, sample size, randomization, allocation concealment, blinding, inclusion criteria, and exclusion criteria.
3. Participants: age, gender, severity of hypertension, diagnostic criteria for hypertension.
4. Interventions: type of control, treatment time, frequency of treatment.
5. Results: primary and secondary outcomes, adverse events, and follow-up.

##### 2.3.3.3 Assessment of bias in the included studies

Two reviewers will independently use the “Risk of Bias” tool - Cochrane Handbook for Systematic Reviews of Interventions V.5.3.5 to conduct quality assessment. Quality will be assessed using the following methods: random sequence generation, random allocation concealment, blind execution methods, result evaluators, results data integrity, selective results reporting, and other biases. Each item will be rated as high risk bias, low risk bias, and uncertain bias. When there is any disagreement, it will be resolved through discussion or consultation with the 3rd author.

##### 2.3.3.4 Measures of treatment effect

The continuous data will be expressed as mean difference (MD) or standard mean difference (SMD) with 95% confidence intervals (CIs), and the dichotomous outcomes will be estimated by the risk ratio (RR) with 95% CIs.

##### 2.3.3.5 Dealing with missing data

If the required data is unclear or not reported in the clinical paper, the reviewer will contact the 1st author or corresponding author of the study by phone, email, or mail to collect the missing data in the data collection form.

##### 2.3.3.6 Assessment of heterogeneity

The heterogeneity of the included studies will be analyzed by Chi-squared test (*a* = 0.1), the value of which is determined by *I*^2^. *I*^2^ < 50% will be considered to contain small heterogeneity of included studies, while *I*^2^ > 50% will be considered to have greater heterogeneity. We will select subgroup analysis and sensitivity analysis to detect sources of possible clinical or methodologic heterogeneity.

##### 2.3.3.7 Assessment of reporting bias

When more than 10 studies are included, a funnel plot will be used to detect report bias.

##### 2.3.3.8 Data synthesis

Data analysis and synthesis will be performed using RevMan V.5.3.5. Continuous data are expressed as MD/SMD, 95% CIs, and the results of the 2 classifications are expressed as RR, 95% CIs. When *I*^2^ < 50%, the fixed effect model is used for analysis; when *I*^2^ > 50%, the random effect model is selected. When significant clinical heterogeneity is observed, researchers can turn to subgroup or sensitivity analysis, or just descriptive analysis. *α* = 0.05 was used to evaluate the meta-analysis.

##### 2.3.3.9 Subgroup analysis

Subgroup analyses will be performed according to different interventions, control measures, and outcome indicators. Adverse reactions will be assessed and tabulated by descriptive techniques. (The number of studies is >10.)

##### 2.3.3.10 Sensitivity analysis

If sufficient studies are included, we will conduct a sensitivity analysis to test the robustness and reliability of the results. Sensitivity analysis focuses on the characteristics or types of research, such as methodologic quality, and examines the effects of overall outcomes by excluding certain low-quality studies or nonblind studies.

##### 2.3.3.11 Grading the quality of evidence

The GRADE profiler software (Version 3.6, The GRADE Working Group) will be used to analyze the quality level of evidence.

##### 2.3.3.12 Ethics and dissemination

Since our research does not include information identifying individuals, there is no need for ethics approval. The study will be published in peer-reviewed journals or related conferences.

## 3 Discussion

Hypertension is the most common chronic disease in the clinic and is an important risk factor for cardiovascular and cerebrovascular diseases.^[14]^ Antihypertensive drugs are the main method for the treatment of hypertension, but due to various limitations such as adverse reactions and complications in clinical use, it is difficult to achieve the desired therapeutic goals. Interventions such as lifestyle behaviors and diet control can be used to lower blood pressure, but these interventions are difficult to adhere to.^[15]^ Endothelial injury caused by hypertension has received increasing attention. TCM has been used as an alternative therapy. Many studies have shown that TCM can effectively lower blood pressure and improve vascular endothelial function in patients with hypertension, reducing the occurrence of complications.^[16,17]^ However, there is no systematic review and meta-analysis of its impact and safety. Therefore, it is necessary to conduct high-quality systematic reviews and meta-analysis. We hope that this systematic review will provide more persuasive evidence for the treatment of essential hypertension with TCM and western medicine. However, there may be some potential shortcomings in the systematic review. First, heterogeneity risks can occur due to different doses, age and severity of hypertension. Second, a small sample may lead to a high risk of bias.

## Author contributions

**Conceptualization:** Weiquan Ren, Li Huang.

**Data curation:** Weiquan Ren, Jiangquan Liao, Jialiang Chen.

**Formal analysis:** Weiquan Ren, Jiangquan Liao, Jialiang Chen.

**Project administration:** Weiquan Ren, Li Huang.

**Supervision:** Li Huang, Zhonghao Li.

**Writing – original draft:** Weiquan Ren.

**Writing – review & editing:** Weiquan Ren, Jiangquan Liao.
